# A Comparative Study on the Degradation Behaviors of Ferroelectric Gate GaN HEMT with PZT and PZT/Al_2_O_3_ Gate Stacks

**DOI:** 10.3390/mi15010101

**Published:** 2024-01-05

**Authors:** Lixiang Chen, Zhiqi Lu, Chaowei Fu, Ziqiang Bi, Miaoling Que, Jiawei Sun, Yunfei Sun

**Affiliations:** School of Electronic and Information Engineering, Suzhou University of Science and Technology, Suzhou 215009, China

**Keywords:** GaN, MIS-HEMT, ferroelectric, reliability, traps

## Abstract

In this paper, the degradation behaviors of the ferroelectric gate Gallium nitride (GaN) high electron mobility transistor (HEMT) under positive gate bias stress are discussed. Devices with a gate dielectric that consists of pure Pb(Zr,Ti)O_3_ (PZT) and a composite PZT/Al_2_O_3_ bilayer are studied. Two different mechanisms, charge trapping and generation of traps, both contribute to the degradation. We have observed positive threshold voltage shift in both kinds of devices under positive gate bias stress. In the devices with a PZT gate oxide, we have found the degradation is owing to electron trapping in pre-existing oxide traps. However, the degradation is caused by electron trapping in pre-existing oxide traps and the generation of traps for the devices with a composite PZT/Al_2_O_3_ gate oxide. Owing to the large difference in dielectric constants between PZT and Al_2_O_3_, the strong electric field in the Al_2_O_3_ interlayer makes PZT/Al_2_O_3_ GaN HEMT easier to degrade. In addition, the ferroelectricity in PZT enhances the electric field in Al_2_O_3_ interlayer and leads to more severe degradation. According to this study, it is worth noting that the reliability problem of the ferroelectric gate GaN HEMT may be more severe than the conventional metal–insulator–semiconductor HEMT (MIS-HEMT).

## 1. Introduction

Gallium nitride(GaN) high electron mobility transistor (HEMT) has been studied over the last decade in consideration of high power and high frequency electronic applications [1,2,3,4,5,6,7,8,9]. Recently, GaN HEMT with ferroelectric insulated gate have attracted a large number of attentions. Of particular interest is the combination of ferroelectric oxides with GaN HEMT due to the strong polarization of GaN-based materials and the switchable polar nature of ferroelectrics, leading to the possibility of tunning the polarization in GaN HEMT with ferroelectric oxides. Many studies focus on achieving E-mode GaN metal–insulator–semiconductor HEMT(MIS-HEMT) by modulating the polarization of AlGaN/GaN with ferroelectric material [10,11,12]. Extensive studies have reported that the growth of ferroelectric oxides on AlGaN/GaN heterostructure could improve the lattice-mismatch problem, the basic electric characteristics of device after modulation of threshold voltage by ferroelectric oxides, or the polarization-modulation behavior between the ferroelectric gate and AlGaN/GaN [13,14]. Similar as the conventional GaN MIS-HEMT, the presence of the dielectric layer would cause threshold voltage drifts due to trapping at the dielectric/III-V interface under positive gate bias stress [15,16,17,18]. Especially for ferroelectric gate GaN MIS-HEMT, the ferroelectric oxide layer may lead to different degeneration effects due to the polarization coupling between the ferroelectric oxide and AlGaN/GaN. However, so far, little attention has been paid on the reliability of the ferroelectric gate GaN MIS-HEMT under electric stress. The degeneration of electric characteristics under electric stress limits the applied voltage at which the ferroelectric gate GaN HEMT can be safely operated.

As Pb(Zr,Ti)O_3_ (PZT) is a typical ferroelectric for the dielectric layer in ferroelectric/AlGaN/GaN HEMT, in this paper, we investigate the roles of charge trapping and trap generation during the positive gate bias stress in PZT/AlGaN/GaN HEMT devices. The threshold voltage, subthreshold, and transconductance are examined before and after the stress test to reveal the different trap effects during the test. As compared, the device with an Al_2_O_3_ interlayer between PZT and AlGaN(PZT/Al_2_O_3_/AlGaN/GaN HEMT) is also investigated, which shows a different degeneration behavior from that without Al_2_O_3_.

## 2. Device Design and Fabrication

**Figure 1:**
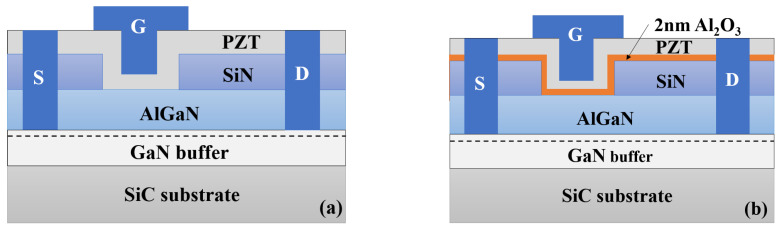
Schematic cross-section of (**a**) the PZT/AlGaN/GaN HEMT and (**b**) the PZT/Al_2_O_3_/AlGaN/GaN HEMT.

The structure diagram of the fabricated ferroelectric gate AlGaN/GaN HEMT is presented in Figure 1. The device was fabricated on SiC substrate by metal organic chemical vapor deposition (MOCVD), containing 180 nm AlN nucleation layer, 3-μm GaN buffer, 1 nm AlN interlayer, and 20 nm Al_0.3_Ga_0.7_N barrier. Device fabrication started with ohmic contact formation by depositing a Ti/Al/Ni/Au (20/160/55/45 nm) metal stack annealed at 840 °C for 30 s in N_2_ ambient, followed by the device isolation using inductively coupled plasma (ICP). The pressure and N_2_ flow during the annealing is one atmosphere (101 kPa) and 2L/min, respectively. The contact resistance of the Ti/Al/Ni/Au metal stack is 0.45 Ω·mm measured by 2-probe linear transmission line method (TLM). Then, the surface passivation was conducted by depositing Si_3_N_4_ using plasma-enhanced chemical vapor deposition (PECVD). The SiN presented under the gate was removed by dry-etching with ICP. PZT was deposited using a pulsed laser deposition (PLD) system at 500 °C. For the device with Al_2_O_3_ interlayer as shown in Figure 1b, prior to a 30 nm PZT ferroelectric layer deposition, a 2 nm Al_2_O_3_ interfacial layer was grown by atomic layer deposition (ALD) and oxidized with O_2_ plasma at 300 °C for 30 min. Finally, Pt was sputtered using a magnetron sputter and lifted off to define the gate electrodes.

## 3. Device Performance and Discussion

**Figure 2:**
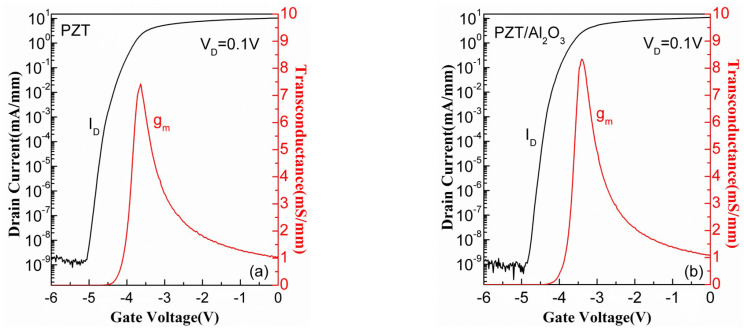
Transfer characteristic of (**a**) the PZT/AlGaN/GaN HEMT and (**b**) the PZT/Al_2_O_3_/AlGaN/GaN HEMT at V_D_ = 0.1 V.

The DC measurements were performed using a Keithley 4200 semiconductor analyzer. In this paper, we focus on the stability of the threshold voltage (V_th_), the maximum transconductance(g_m_), and the subthreshold swing (S.S) of the devices with and without a Al_2_O_3_ interlayer. To compare the characteristics of the devices before and after the stress test, the transfer characteristics of both kinds of fresh devices (PZT GaN HEMT and PZT/Al_2_O_3_ GaN HEMT) were measured, as presented in Figure 2. Drain voltage was applied with 0.1 V to measure the transconductance and subthreshold in the linear region, which decreases the influence of the drain electric field on the gate. The peak gm of the two types of devices was 7.5 mS/mm and 8.3 mS/mm, respectively.

**Figure 3:**
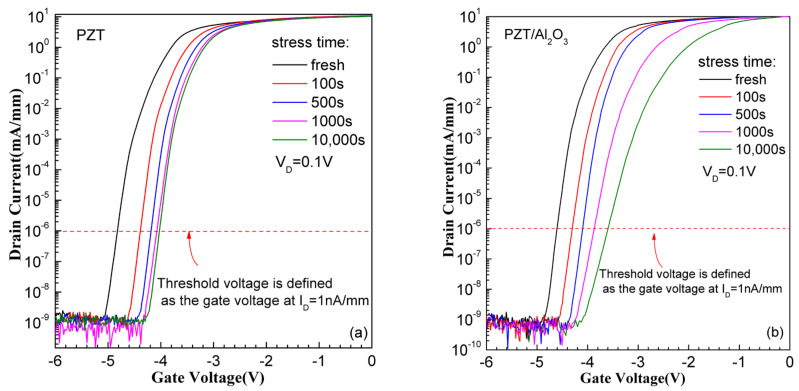
The I_D_-V_GS_ curves of (**a**) the PZT/AlGaN/GaN HEMT and (**b**) the PZT/Al_2_O_3_/AlGaN/GaN HEMT after positive gate bias stress with different stress time.

During the test, positive gate bias stress was applied with 15 V with drain and source grounded, and the stress time (t_stress_) was set from 10 s to 10,000 s. The transfer characteristics of both kinds of devices were measured during the positive stress to monitor the evolution of V_th_, g_m_ and S.S with stress time. Figure 3 shows the I_D_-V_GS_ curves of PZT GaN HEMT and PZT/Al_2_O_3_ GaN HEMT in several typical stress times. The threshold voltage is defined as the gate voltage at I_D_ = 1 nA/mm. As can be seen, the threshold voltages of both kinds of devices positively shift with the increase in stress time. For PZT GaN HEMT, the Vth shifting tend to saturation with stress time increased to 1000 s, while Vth shifting of PZT/Al_2_O_3_ GaN HEMT is still obvious when the stress time reaches 10,000 s. The Vth shift during positive gate bias stress indicates electron trapping in the gate stack. 

However, it is worth pointing out that the S.S is almost no changing for the PZT GaN HEMT as shown in Figure 3a, which means no traps generation during the stress in PZT GaN HEMT. As for the PZT/Al_2_O_3_ GaN HEMT, the S.S degrades when stress time increase to 1000 s, indicating the generation of traps (interface or border) according to the formula for the S.S [19]. These generated traps cause the positive shift of V_th_ when the stress time increase to 1000 s for the PZT/Al_2_O_3_ GaN HEMT.

**Figure 4:**
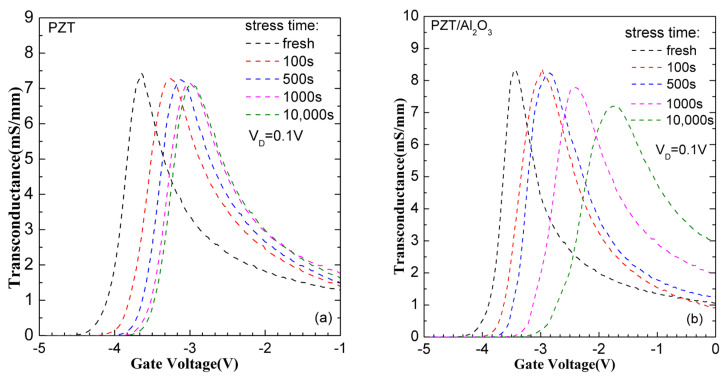
Transconductance curves of (**a**) the PZT/AlGaN/GaN HEMT and (**b**) the PZT/Al_2_O_3_/AlGaN/GaN HEMT after positive gate bias stress with different stress time.

Figure 4 presents the transconductance curves extracted from Figure 2. It is easy to see that the g_m_ drops slightly for the PZT GaN HEMT while the g_m_ decreases obviously when the stress time reaches 1000 s for the PZT/Al_2_O_3_ GaN HEMT. The obvious drop of g_m_ during the stress suggests that the generated traps have a significant influence on the mobility of the PZT/Al_2_O_3_ GaN HEMT. The charge-trapping process mainly occurs near the oxide/AlGaN interface which is close to the channel because of its influence on channel mobility.

**Figure 5:**
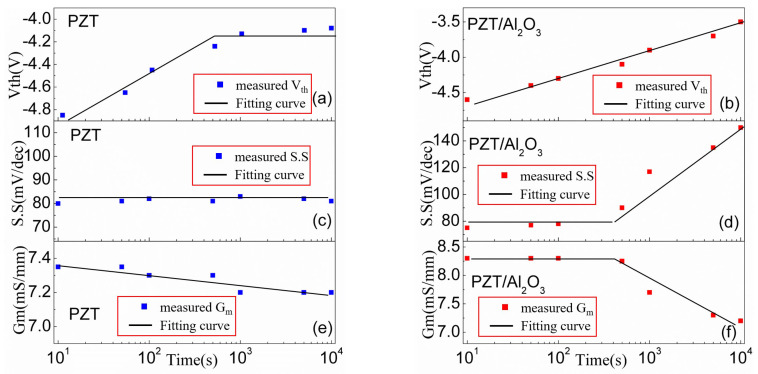
Stress time evolution of Vth, S.S, and g_m_ of (**a**,**c**,**e**) the PZT/AlGaN/GaN HEMT and (**b**,**d**,**f**) the PZT/Al_2_O_3_/AlGaN/GaN HEMT.

In order to identify the different trends of V_th_, S.S, and g_m_ for both kinds of devices during the stress applied, Figure 5 summarizes the evolution of V_th_, S.S, and g_m_ over the stress time. For the PZT GaN HEMT, the Vth increases linearly with stress time before 600s, and then increases very slightly with stress time, as can be seen in Figure 5a. The S.S is almost stable at around 80~85 mV/dec during the stress test as shown in Figure 5c. In Figure 5e, the g_m_ drops slightly with stress time increases, indicating that the electron trapping just has a slight impact on the mobility of the device. Since g_m_ drops slightly and no S.S degeneration during the stress test, it can be concluded that the V_th_ positive shift is mainly due to the electron trapping in the oxide for the PZT GaN HEMT. Besides, due to there is no generated traps during the stress test, the V_th_ shift tends to saturation when the stress time increases after 600 s. For the PZT/Al_2_O_3_ GaN HEMT, the V_th_ increases with stress time even in long-term stress region as shown in Figure 5b, which is different from the PZT GaN HEMT. When the stress time is less than 500 s, the S.S and g_m_ are stabilized at about 80 mV/dec and 8.3 mS/mm, respectively. The S.S starts to increase, and g_m_ starts to drop when stress time is above 500 s. This suggests that new trap generation takes place for the PZT/Al_2_O_3_ GaN HEMT when stress time is greater than a certain level. According to the discussion above, we find that the PZT/Al_2_O_3_ GaN HEMT is easier to degradation under positive gate bias stress than the PZT GaN HEMT. 

**Figure 6:**
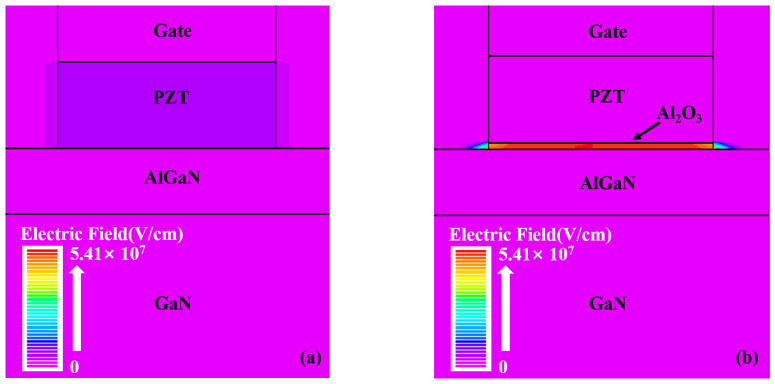
Electric field simulation of (**a**) the PZT/AlGaN/GaN HEMT and (**b**) the PZT/Al_2_O_3_/AlGaN/GaN HEMT at V_G_ = 15 V.

To clarify the reason for the new trap generation of the PZT/Al_2_O_3_ GaN HEMT in our stress test, we simulated the electrical field distribution under the gate of both kinds of devices with V_G_ = 15 V by Silvaco Technology Computer Aided Design (TCAD). In our simulation, the dielectric constant of PZT and Al_2_O_3_ was set to 500 and 9, respectively. As shown in Figure 6, the electrical field under the gate stacks mainly concentrate on Al_2_O_3_ interlayer for the PZT/Al_2_O_3_ GaN HEMT, which is owing to the large difference in dielectric constants between PZT and Al_2_O_3_. The strong electric field in the Al_2_O_3_ interlayer leads to the generation of new traps during the stress test, which degrades the S.S and g_m_ of devices. For the PZT GaN HEMT, the electric field in the gate oxide layer is relatively weak and therefore not so easy to degrade.

**Figure 7:**
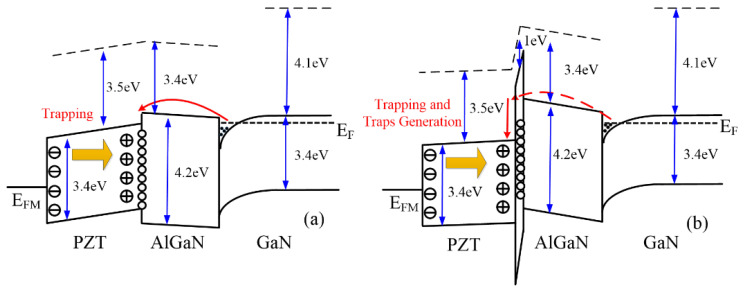
Band diagrams under the gate of (**a**) the PZT/AlGaN/GaN HEMT and (**b**) the PZT/Al_2_O_3_/AlGaN/GaN HEMT at V_G_ = 15V.

Figure 7 presents the band diagram to illustrate the underlying reason for the trapping behavior of both kinds of devices during the positive gate stress test. The direction of polarization in PZT is toward to substrate due to the applied gate voltage is positive. For the PZT GaN HEMT, there are some trap states at the PZT/AlGaN interface (interface states) or in the PZT close to the interface (border traps) due to the lattice mismatch between PZT and AlGaN [13]. Therefore, as shown in Figure 7a, the electrons from AlGaN/GaN channel are trapped at the PZT/AlGaN interface with the positive gate voltage, which leads to the V_th_ positive shift as mentioned above. When the trap states below the fermi level are all filled, the V_th_ shifting approaches saturation. 

There are two stages of electrons trapping for the PZT/Al_2_O_3_ GaN HEMT. At the first stage, similar as the PZT GaN HEMT, electrons trapped at Al_2_O_3_/AlGaN interface or in Al_2_O_3_ interlayer with the positive gate voltage, causing the positive V_th_ shift. At the second stage, as shown in Figure 7b, electrons tunnel through the Al_2_O_3_ interlayer and enter the PZT. Owing to the conduction band difference between Al_2_O_3_ and PZT, the tunneling electrons start to lose energy in PZT and the lost energy from these electrons could be used to generate traps at Al_2_O_3_ or near the PZT/Al_2_O_3_ interface. Moreover, a strong electric field in Al_2_O_3_ makes a large conduction band bending, which leads to the tunnel electrons losing more energy and cause more severe generation of traps. These new trap states may be filled by electrons from the channel, which not only leads to the V_th_ positive shift continually but also causes the S.S and g_m_ degradation with the increase in stress time. In addition, the ferroelectricity in PZT could also enhance the electric field in the Al_2_O_3_ interlayer (indicated by the arrow in Figure 7b) compared with the case that gate stacks are non-ferroelectric [19]. Such a high electric field would cause more severe degradation of the devices compared with the conventional MIS-HEMT. It is also worth pointing that some research shows utilizing oxides as interlayer can mitigate the lattice mismatch between PZT and AlGaN, but it also makes the device more susceptible to degradation. Furthermore, the ferroelectricity in PZT makes the degradation more severe.

## 4. Conclusions

In conclusion, this paper studied the positive gate bias stress of the ferroelectric gate GaN HEMT with PZT and PZT/Al_2_O_3_ as gate dielectric. The degradation mechanisms of the PZT GaN HEMT and the PZT/Al_2_O_3_ GaN HEMT are compared. For the PZT GaN HEMT, a positive V_th_ shift is caused by the electron trapping in pre-exiting oxide trap in the early stage, then the V_th_ tends to saturation with stress time increase. The S.S and g_m_ degrade slightly with stress time. For the PZT/Al_2_O_3_ GaN HEMT, the V_th_ positive shift is due to the electron trapping in pre-exiting oxide trap and the generation of the new traps. The generated traps cause the obvious degradation of the S.S and gm. Owing to the large difference in the dielectric constants between PZT and Al_2_O_3_, the strong electric field in the Al_2_O_3_ interlayer makes the PZT/Al_2_O_3_ GaN HEMT easier to degrade. In addition, the ferroelectricity in PZT enhances the electric field in Al_2_O_3_ interlayer and leads to more severe degradation. Therefore, it is worth noting that the reliability problem of the ferroelectric gate GaN HEMT may be more severe than the conventional GaN MIS-HEMT. Using the ferroelectric oxide with a relatively small dielectric constant in gate stacks may mitigate the degradation in the real applications.

## Data Availability

Data are contained within the article.
